# A single bout of resistance or high-intensity interval training increases anti-cancer myokines and suppresses cancer cell growth *in vitro* in survivors of breast cancer

**DOI:** 10.1007/s10549-025-07772-w

**Published:** 2025-07-03

**Authors:** Francesco Bettariga, Dennis R. Taaffe, Cristina Crespo-Garcia, Timothy D. Clay, Mauro De Santi, Giulia Baldelli, Sanjeev Adhikari, Elin S. Gray, Daniel A. Galvão, Robert U. Newton

**Affiliations:** 1 Exercise Medicine Research Institute, Edith Cowan University, Joondalup, Australia; 2 School of Medical and Health Sciences, Edith Cowan University, Joondalup, Australia; 3 Department of Oncology, St John of God Subiaco Hospital, Perth, WA Australia; 4 Department of Biomolecular Sciences, Section of Hygiene, University of Urbino Carlo Bo, Urbino, Italy; 5 Centre for Precision Health, Edith Cowan University, Joondalup, Australia; 6 School of Human Movement and Nutrition Sciences, University of Queensland, St Lucia, Australia

**Keywords:** Breast cancer, Resistance training, High intensity interval training, Myokine, Cancer cell

## Abstract

**Purpose:**

Breast cancer is the leading cause of cancer-related death in women, highlighting the need for strategies to mitigate recurrence and mortality. We examined the effects of a single bout of resistance training (RT) versus high-intensity interval training (HIIT) on anti-cancer myokines *and *in vitro cancer cell suppression.

**Methods:**

Thirty-two survivors of breast cancer were randomly allocated to a single bout of RT (n = 16) or HIIT (n = 16). Blood was collected before, immediately post (0P) and 30 min post (30P) exercise. We measured serum levels of decorin, interleukin 6 (IL-6), secreted protein acidic and rich in cysteine (SPARC), and oncostatin M (OSM) and cell growth of MDA-MB-231 cells in vitro using real time cellular analysis at each time point.

**Results:**

Decorin, IL-6, and SPARC significantly increased (9 to 47%, *p* < 0.05) from baseline to 0P in both groups. IL-6 remained elevated in both groups at 30 min post-intervention (30P), while OSM levels were elevated only in the RT group at 30P. Between groups, IL-6 was significantly increased in HIIT at 0P (*p* = 0.001). Cancer cell growth was significantly reduced at 0P and 30P compared to baseline in RT (20 to 21%, *p* < 0.05) and HIIT (19 to 29%, *p* < 0.05), with significantly greater effects on MDA-MB-231 cell growth reduction in favour of HIIT at 0P (*p* = 0.001).

**Conclusion:**

A single bout of RT or HIIT can increase levels of anti-cancer myokines and reduce the growth of MDA-MB-231 cells in vitro in survivors of breast cancer, potentially contributing to a lower risk of recurrence. This highlights the importance of exercise as a treatment with promising anti-cancer effects.

## Introduction

Breast cancer is a major global health concern contributing substantially to both morbidity and mortality rates [1]. In 2022 alone, 2.3 million new cases of breast cancer were diagnosed worldwide with approximately 660,000 deaths, making breast cancer the most diagnosed and the leading cause of cancer-related death in women [1]. Furthermore, for survivors of breast cancer, recurrence rates remain relevant even years after treatment, with estimates ranging from 10 to 30% depending on tumor subtype, stage at diagnosis, menopausal status, and time since treatment completion [2]. This highlights the need for advancements in prevention and treatment aimed specifically to mitigate the risk of recurrence and mortality [3, 4].

Exercise has emerged as a therapeutic intervention in the management of breast cancer and is extensively supported by international guidelines [5, 6]. Indeed, a robust body of evidence now exists on the safety and effectiveness of exercise as medicine, either during or post cancer treatments for breast cancer (e.g., chemotherapy, hormone therapy, targeted therapy), to improve various health-related cancer outcomes (e.g., fatigue, quality of life, cardiorespiratory fitness, neuromuscular strength, body weight and body composition) [7–9]. Furthermore, exercise is also associated with ≈ 20% lower risk for recurrence and mortality in patients with breast cancer [10, 11]. Notably, higher levels of physical fitness (i.e., muscle strength and cardiorespiratory fitness) are associated with a 31 to 46% reduced risk of all-cause mortality in patients with cancer [12]. Furthermore, exercise carries little to no risk of morbidity, in comparison to many other therapeutic interventions (e.g., chemotherapy, surgery). The underlying biological mechanisms linking exercise to enhanced survival are not fully understood, and more research is needed.

Skeletal muscle has been recognized for its function as an endocrine organ [13, 14], capable of secreting signalling molecules known as myokines. Myokines are a subset of cytokines or peptides that are produced and released by muscle cells at rest (e.g. irisin, decorin) and in response to muscular contractions (e.g., interleukin 6 [IL-6], secreted protein acidic and rich in cysteine [SPARC], oncostatin M [OSM]) to exert either paracrine, autocrine, or endocrine effects [15, 16]. Among the wide array of positive effects on body organs (e.g., bone, brain, liver, skeletal muscle, adipose tissue, etc.), preclinical and translational in vitro studies have shown the potential role of myokines (e.g., IL-6, OSM, decorin) to suppress cancer cell growth in different cancer cell lines [17–20]. Briefly, IL-6 has been shown to induce apoptosis and inhibit proliferation in certain breast cancer subtypes [21]; OSM is associated with promoting cancer cell dormancy and reducing metastatic potential [22]; decorin interferes with tumor growth by modulating the tumor microenvironment and inhibiting receptor tyrosine kinases [23]; and SPARC plays a role in inhibiting tumor progression by regulating cell adhesion, migration, and extracellular matrix remodeling [24]. Experimental studies in healthy individuals and cancer patients show that serum collected after a single exercise bout suppresses cancer cell growth, proliferation, and viability, while increasing apoptosis in various cancer cell lines, including breast cancer [19, 25–28]. However, only two studies have investigated the acute effects on cancer cells in patients with breast cancer [26, 29], while others were conducted in healthy individuals [25, 30]. This is of utmost importance because breast cancer and treatments (e.g., hormone therapy) alter several physical and physiological components and may impair a patient’s ability to adapt to the morphological and metabolic changes induced by exercise [19, 31], meaning that more research in patients as well as survivors of breast cancer is needed [19].

In regard to exercise training, the aforementioned acute studies adopted different forms of aerobic training (AT), including moderate intensity continuous training (MICT) or high intensity interval training (HIIT), as well as a combined approach encompassing both AT and resistance training (RT) [25, 26, 29, 30]. However, to date, research in this field is limited to a single-arm study design [25, 26, 29, 30], and no studies have explored the distinct effects of different exercise modes, namely RT and HIIT, on myokine expression and their suppressive effects on breast cancer cells [16, 19]. This is important, as such findings could inform targeted exercise prescriptions to reduce cancer growth and recurrence in breast cancer survivors [19]. Therefore, it is critical to investigate the acute effects of different exercise modes on the expression of anti-cancer myokines, such as IL-6 [32], OSM [33], decorin [34], and SPARC [35], and their effects on breast cancer cell growth (i.e., MDA-MB-231) [16, 19]. Therefore, the aims of the current study are: 1) to examine the acute effects of two different exercise modes on myokine expression and cancer cell suppression in survivors of breast cancer; 2) to determine whether RT or HIIT induce different responses in myokine levels and cancer cell suppression.

## Methods

This was a study comparing a single bout of RT vs HIIT on myokine expression and exercise-conditioned serum on in vitro cell suppression in breast cancer survivors. The single bout of exercise was performed before commencing a 12-week exercise intervention which has been previously described [8]. Ethical approval was obtained from the Edith Cowan University Human Research Ethics Committee (ID: 2023–04617-BETTARIGA) and the trial was registered on ANZCTR (ID: ACTRN12624000820505).

### Participants, recruitment, and allocation

Eligible participants were women initially diagnosed with stage I–III breast cancer who had completed primary treatment (e.g., surgery, chemotherapy, or radiation therapy) at least four months prior, had a body mass index (BMI) between 18.5 and 35 kg/m^2^, and were medically cleared for exercise. Patients continued on endocrine therapy when prescribed by their treating specialist. Exclusion criteria were any absolute contraindication to exercise, sustained vigorous exercise in the past 3 months (i.e., ≥ 150 min of moderate or ≥ 75 min of high-intensity AT, or ≥ 2 RT sessions per week over the past three months), a life expectancy of less than 12 months, and pregnancy or lactation. Recruitment occurred at the Exercise Medicine Research Institute, Edith Cowan University in Perth, Western Australia, between 1st October 2023 and 1st July 2024. All participants provided written informed consent before enrolment and were randomly assigned, using REDCap software, to RT or HIIT in a ratio of 1:1.

### Outcome measures

#### Blood assessment and analysis

A 16 mL blood sample was collected at three time points: at baseline (following a 2-h fast), immediately after the acute exercise session (0P), and 30 min post-exercise (30P). Blood samples were immediately processed to separate serum for subsequent analysis and stored at -80⁰ Celsius.

Serum levels of decorin, IL-6, OSM, and SPARC were analyzed in duplicate using commercially available enzyme-linked immunosorbent assay (ELISA) kits (Human ELISA Kit, Abcam, Cambridge, UK) according to the manufacturer’s instructions. The assay sensitivities were 1.5 pg/mL for decorin, 0.97 pg/mL for IL-6, 1.2 pg/mL for OSM, and 125 pg/mL for SPARC. The intra- and inter-assay coefficients of variation for all assays were below 15%, indicating acceptable reliability.

#### Real time cellular analysis

The human triple negative breast cancer cell line MDA-MB-231 was cultured in DMEM containing 10% foetal bovine serum and routinely passaged at ≈ 80% confluence. The growth of MDA-MB-231 cells was assessed using a Real-Time Cellular Analysis (RTCA) system, xCELLigence DP unit and E-plate (ACEA Bioscience, CA, USA) in the presence of serum from each participant at three different time points (baseline, 0P, and 30P). Each well of the E-plate was seeded with 10,000 MDA-MB-231 cells in 100 μl of DMEM. Following 16 h of starvation (MDA-MB-231 incubated with DMEM only), an additional 100 μl of growth media (DMEM) containing 40% human serum from each participant and time point (resulting in a final concentration of 20%) was added to each well. The growth rate of MDA-MB-231 cells in response to individual serum samples was assessed in duplicate. The plates were then incubated for 72 h, with cell growth monitored every 15 min in the unit of Cell Index (Cell Index × Time) to calculate the area under the curve (AUC).

#### Acute exercise protocol

After randomization to RT or HIIT, participants undertook muscle strength testing for the leg press and chest press using the 1-repetition maximum (1RM), which involves determining the maximum weight a participant can lift for a single repetition of an exercise [8]. Participants then completed two familiarization sessions and one week later performed either a single bout of RT or HIIT lasting approximately 45 min, in addition to a 10-min warm-up. Participants allocated to RT performed 8 repetitions for 5 sets of exercises for the major muscle groups, including chest press, seated row, shoulder press, lat pulldown, leg press, leg extension, leg curl, and lunges. Intensity was adjusted according to participants’ tolerance in order to achieve an rating of perceived exertion (RPE) of 7 to 9 (using the 1 to 10 RPE scale [36]) or > 80% 1RM [37]. A 60- to 120-s rest period was provided between sets. For time efficiency and to minimize fatigue, exercises were alternated between the upper and lower body (e.g., chest press then leg press).

Participants allocated to the HIIT performed 7 bouts of 30 s at high intensity, with active recovery of 30 s, for 4 sets on at least three of the following exercise machines: stationary cycle, treadmill, rower, and cross-trainer. Intensity was adjusted according to participants’ tolerance in order to achieve a RPE of 7 to 9 (using the 1 to 10 RPE scale [36]) or 70 to 90% of estimated HRmax (220 – age) for the high intensity bouts. During the active recovery, participants were required to maintain a slow pace at an RPE of ≈ 3. A 180-s rest period was provided between sets.

### Statistical analysis

We considered a between-group difference of 1 standard deviation in our outcomes of interest to be clinically meaningful (i.e., an effect size of 1.0). Based on this, a sample size calculation was conducted from the original 12-week intervention study [8], indicating that 16 participants per group (32 in total) would provide 80% power to detect such a difference at an alpha level of 0.05 (two-tailed). Although the current study reports on an acute, secondary outcome, the same sample size is retained, and remains adequately powered given the nature of the design and the absence of drop-out. Normality of the distribution was assessed using the Shapiro–Wilk test. When normality assumptions were violated, non-parametric tests were used. Repeated measures ANOVA or Friedman test were used to examine within-group changes in myokine levels and cancer cell suppression. Post-hoc analyses were conducted using Bonferroni-corrected pairwise comparisons for ANOVA or Wilcoxon signed-rank test for Friedman, with statistical significance set at *p* < 0.0167 for multiple comparisons, when significant overall effects were found. Between-group differences were analyzed using Generalized Estimating Equations for non-normally distributed data, with pairwise contrasts conducted when interactions were significant. All statistical analyses were performed using R software (v4.4.2, The R Foundation, Vienna, Austria) with significance set at *p* < 0.05.

## Results

**Fig. 1:**
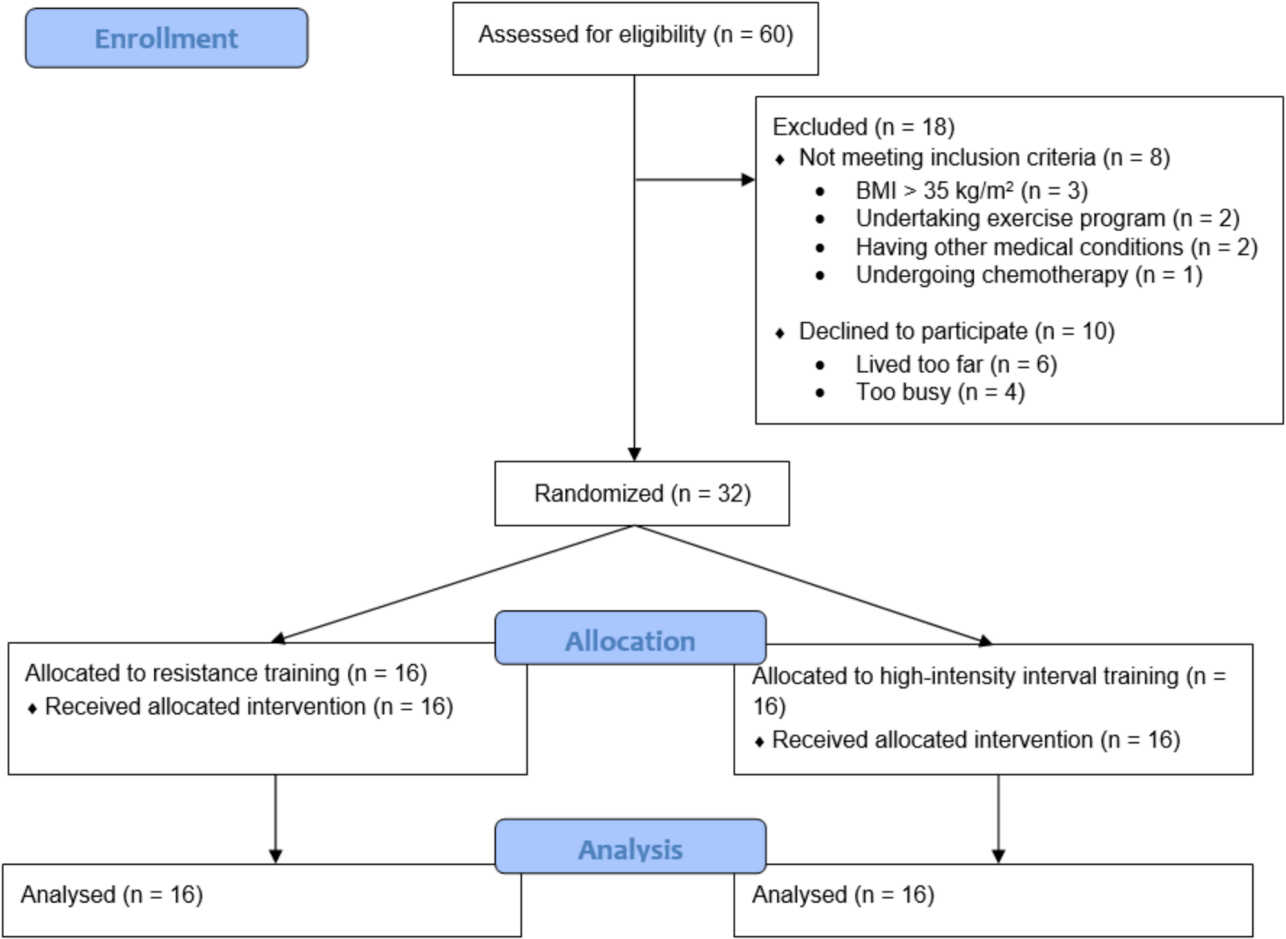
Consolidated Standards of Reporting Trials (CONSORT) diagram

Sixty breast cancer survivors were assessed for eligibility, with 32 recruited and randomized to RT or HIIT (Fig. 1). Baseline demographic, clinical, body composition, and physical fitness characteristics of the participants are reported in Table 1. Briefly, participants had a mean age of 58.6 years ± 8.6, a mean BMI of 27.9 kg/m^2^ ± 5.1, and an average time since diagnosis of 29.3 months ± 15.6. The distribution of disease stages was as follows: 34% at stage I, 41% at stage II, and 25% at stage III. Baseline characteristics, including myokine concentrations and AUC, were comparable between groups as previously reported [8], and no major adverse events were reported or observed during the single bout of exercise.

**Table 1:** Participant characteristics

		RT (n = 16)	HIIT (n = 16)
*Demographic*			
Age (years)		60.8 ± 8.5	56.2 ± 8.8
Body weight (kg)		77.2 ± 12.5	79.6 ± 20.5
Height (cm)		165.6 ± 7.3	166.8 ± 6.7
BMI (kg/m2)		27.9 ± 4.4	27.9 ± 5.6
Time since diagnosis (months)		27.1 ± 8.9	31.5 ± 20.7
Cancer stage (%)	1	44	25
	2	37	44
	3	19	31
Undertaking hormone therapy (%)		63	81
Previous treatments (%)	Chemotherapy	50	94
	Radiation therapy	50	69
	Surgery	100	100
Postmenopausal status (%)		70	64
*Body composition*			
Lean mass (kg)		42.5 ± 5.1	43.1 ± 8.8
Lean mass (%)		55.5 ± 4.8	55.04 ± 5.5
Fat mass (kg)		32.5 ± 8.3	34.55 ± 12.1
Fat mass (%)		41.5 ± 5.2	42.3 ± 5.8
Appendicular lean mass (kg/m2)		6.3 ± 0.7	6.2 ± 1.1
VAT (g)		524.9 ± 280.4	537.3 ± 240.6
*Physical fitness*			
Chest press (kg)		20.1 ± 5.9	22.2 ± 6.4
Leg press (kg)		65.6 ± 26.8	80.3 ± 19.3
VO2max (ml/min/kg)		24.2 ± 3.7	25.2 ± 4.1

Legend:* RT* Resistance training,* HIIT* High intensity interval training, *cm*  Centimetres,* kg* Kilograms,* g * Grams, * %*  Percentage,* VAT* Visceral adipose tissue,* VO2max* Maximal oxygen uptake

### Myokine expression

**Fig. 2:**
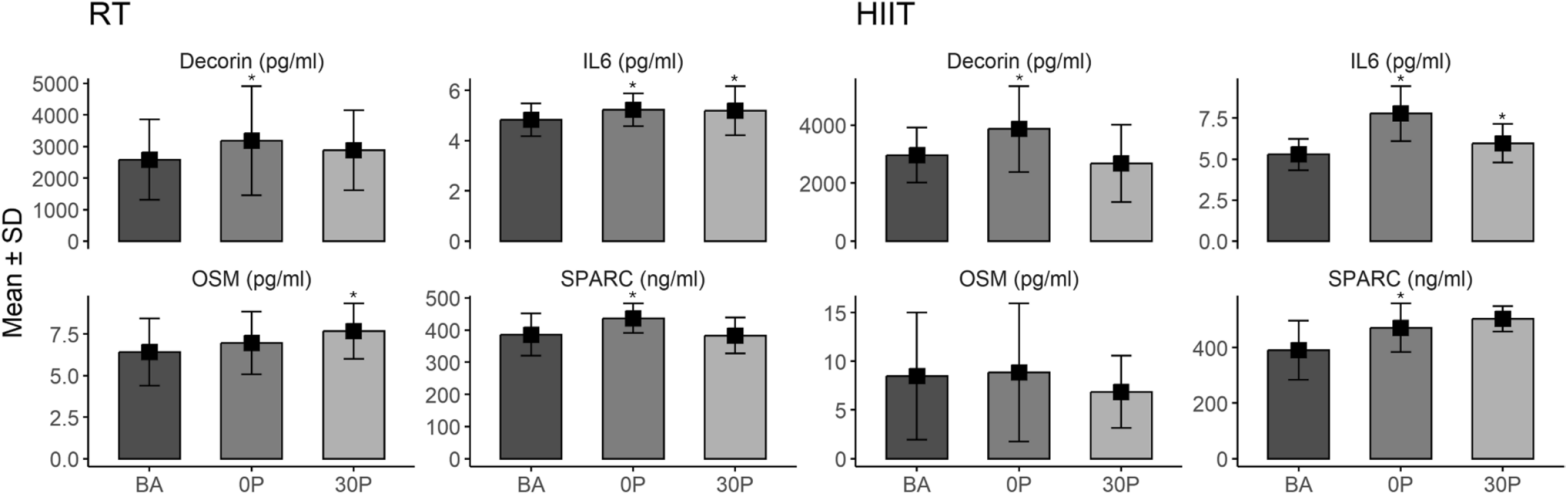
Myokine levels in resistance training and high intensity interval training groups. Legend. pg = picogram; ng = nanogram; BA = baseline; 0P = immediately post exercise; 30P = 30 min post exercise; RT = resistance training; HIIT = high intensity interval training; * = *p* < 0.0167 compared to baseline

Within and between group changes of serum myokine levels at baseline, 0P, and 30P are reported in Table 2 and Fig. 2. When examining the between group changes, there was a statistically significant effect only for IL-6 (*p* = 0.001) at 0P, with a greater increase in favour of HIIT.

**Table 2:** Myokine levels and cell index in resistance training and high intensity interval training groups

	Within-group changes						Between group p-value	
	RT			HIIT				
	Baseline	0P	30P	Baseline	0P	30P	0P	30P
Decorin (pg/ml)	2581.78 ± 1275.7	3187.72 ± 1728.94 *	2882.05 ± 1265.57	2963.46 ± 948.35	3864.27 ± 1480.69 *	2677.44 ± 1329.64	1	1
IL-6 (pg/ml)	4.82 ± 0.65	5.23 ± 0.65 *	5.19 ± 0.98 *	5.29 ± 0.96	7.78 ± 1.68 *	5.97 ± 1.18 *	0.001	0.588
OSM (pg/ml)	6.41 ± 2.03	6.96 ± 1.89	7.66 ± 1.66 *	8.47 ± 6.53	8.84 ± 7.08	6.85 ± 3.71	1	1
SPARC (ng/ml)	385.44 ± 65.70	436.19 ± 45.93 *	382.85 ± 55.43	390.17 ± 106.61	471.32 ± 87.36 *	503.41 ± 45.96	1	1
AUC (Cell Index*Time)	129.22 ± 9.43	94.22 ± 4.74 *	98.22 ± 4.57 *	186.74 ± 15.51	134.97 ± 6.62 *	119.93 ± 6.32 *	0.001 ^	0.098

Legend: *pg*  Picogram, *ng* nanogram, *AUC* Area under the curve, *0P*  Immediately post exercise, *30P*  30 min post exercise, *RT*  Resistance training, *HIIT*  High intensity interval training; * = *p* < 0.0167 compared to baseline. Data are reported as absolute values: mean ± SD for myokines and mean ± SE for AUC

For RT, there were significant changes at repeated time points for decorin (*p* = 0.003), IL-6 (*p* = 0.001), OSM (*p* = 0.001), and SPARC (*p* = 0.001). There were significant increases in decorin (23%, *p* = 0.005), IL-6 (9%, *p* = 0.001), and SPARC (15%, *p* = 0.004) at 0P. There were also significant increases in IL-6 (7%, *p* = 0.002) and OSM (23%, *p* = 0.001) at 30P compared to baseline.

For HIIT, there were significant changes at repeated time points for decorin (*p* = 0.001), IL-6 (*p* = 0.001), and SPARC (*p* = 0.007). There were significant increases in decorin (30%, *p* = 0.001), IL-6 (47%, *p* = 0.001), and SPARC (26%, *p* = 0.004) at 0P. There was also a significant increase in IL-6 (13%, *p* = 0.002) at 30P compared to baseline.

### Real-time cellular analysis

**Fig. 3:**
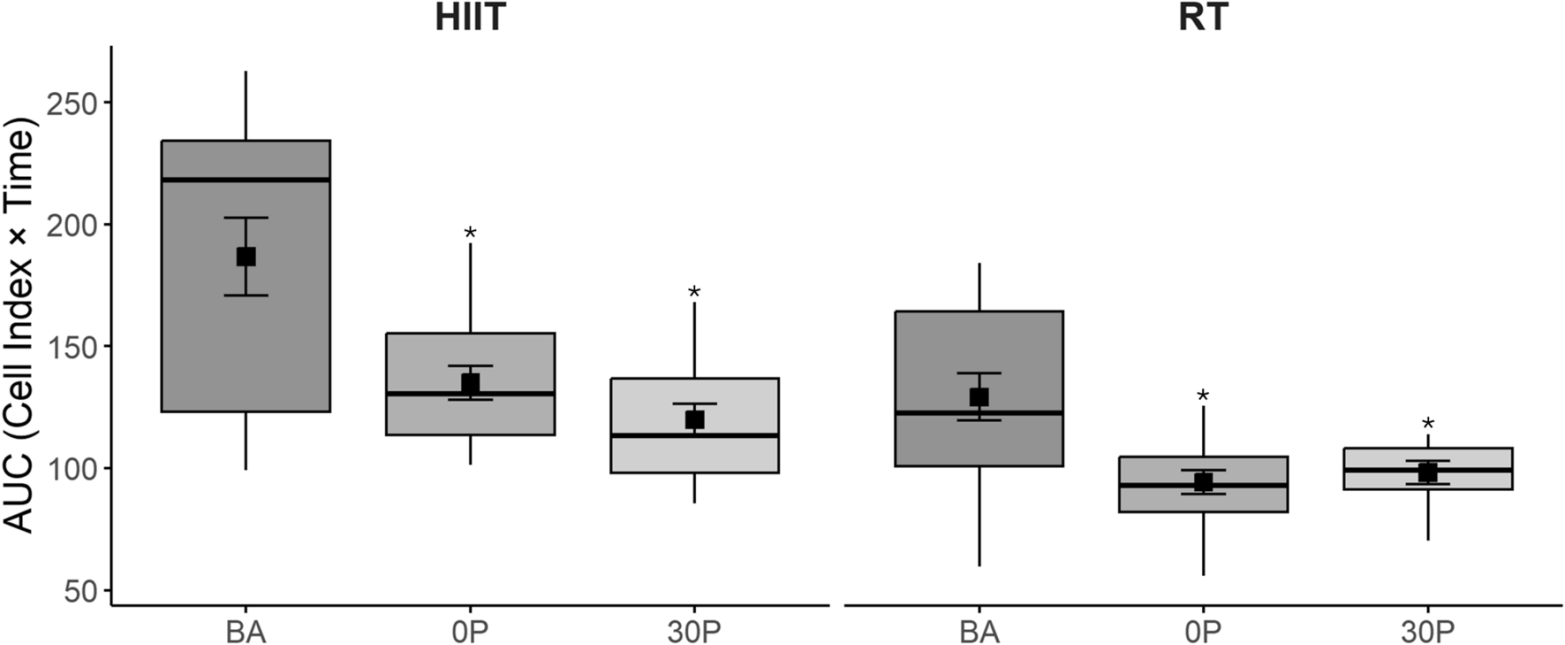
Area under the curve in resistance training and high intensity interval training groups. Legend. RT = resistance training; HIIT = high intensity interval training; BA = baseline; 0P = immediately post exercise; 30P = 30 min post exercise; * = *p* < 0.0167 compared to baseline. Boxes indicate the interquartile range (IQR), from the 25th percentile (Q1) to the 75th percentile (Q3). The horizontal line inside the box represents the median (Q2). The black squares inside the boxes denote the mean, and error bars represent the SE

Within and between group changes of AUC for MDA-MB-231 cells at baseline, 0P, and 30P are reported in Table 2 and Fig. 3. When examining the between group changes in the AUC (0 to 72 h), there was a statistically significant effect for the AUC (*p* = 0.001) only at 0P, with a greater reduction in favour of HIIT.

For RT, there were significant changes at repeated time points for the AUC (*p* = 0.001). There were significant reductions in MDA-MB-231 cell growth (-21%, *p* = 0.001) at 0P and (-19%, *p* = 0.002) at 30P compared to baseline.

For HIIT, there were significant changes at repeated time points for the AUC (*p* = 0.003). There were significant reductions in MDA-MB-231 cell growth (-20%, *p* = 0.006) at 0P and (-29%, *p* = 0.001) at 30P compared to baseline.

## Discussion

The aim of this study was to examine the single bout effects of two different exercise modes on anti-cancer myokine expression and MDA-MB-231 cell suppression in survivors of breast cancer. Two key findings were observed. First, serum levels of decorin, IL-6, and SPARC significantly increased (9 to 47%) from baseline to immediately post exercise in both RT and HIIT groups. IL-6 remained elevated in both groups, while OSM remained elevated in the RT group 30 min post exercise. However, between groups the only significant difference observed was in favour of HIIT for IL-6 immediately post exercise. Second, cancer cell growth was significantly reduced in both groups immediately after as well as 30 min post exercise compared to baseline by 20 to 21% and 19 to 29%, respectively, with significantly greater inhibitory effects on MDA-MB-231 cell growth in favour of HIIT immediately post exercise. Our findings demonstrate that both RT and HIIT elicit acute changes in circulating myokines and reduced cancer cell growth, which may contribute to proposed biological pathways involved in cancer control; however, further research is needed to determine the long-term relevance of these responses for disease recurrence in breast cancer survivors.

Although several preclinical and clinical studies investigated the acute effects of exercise on myokines (e.g., IL-6, OSM, decorin, and SPARC [38, 39]) to determine their potential tumour suppressive role [19], the majority of these studies were conducted in animal models or healthy individuals, limiting their clinical application in the oncology setting. Indeed, cancer and the related treatments may alter several physical (e.g., low muscle mass/strength and cardiorespiratory fitness) and physiological components (e.g., reduced immune and metabolic function), limiting patient’s adaptation to changes induced by exercise [19, 31]. To date, only Dethlefsen et al. [26] examined the acute effects of a 2-h bout of combined exercise training (i.e., RT and HIIT) at moderate to high intensity on IL-6 and IL-8 levels in patients with breast cancer undergoing chemotherapy. They observed an increase of 20 to 110% for these myokines and when applying the exercise-conditioned serum to MCF-7 and MDA-MB-231 cells in vitro, significant reductions in cancer cell viability by ≈ 9% were observed.

Interestingly, although it is well known that a single bout of exercise can alter myokine concentrations [16, 19], it is yet to be determined whether RT or HIIT can exert different responses of myokines in patients with cancer. It is important to clarify that the acute increases in myokines (e.g., IL-6) observed post-exercise represents a beneficial and transient response [19]. However, the long-term implications of these changes, particularly in relation to cancer recurrence, warrant further investigation, as sustained elevations in IL-6 have been associated with chronic inflammation and may promote tumor progression in other contexts [9]. It should also be noted that participant characteristics, such as baseline fitness levels, body composition, and ongoing therapy, may influence the magnitude of the myokine response. Notably, differences in chemotherapy exposure between groups could also have impacted the results, as chemotherapy may alter muscle function, immune responses, and circulating biomarkers. However, this is yet to be determined, and more research is required. For this reason, our findings are of relevance as they expand the knowledge pertaining to the effects of different exercise modes on serum concentrations of myokines in survivors of breast cancer [18, 19]. We observed significant changes in both groups for decorin, IL-6, and SPARC in the serum collected immediately after exercise compared to baseline. Furthermore, elevations in IL-6 and OSM were also noted 30 min post exercise. Interestingly, survivors of breast cancer in both groups had similar increases in myokine levels, apart from a greater increase in IL-6 in favour of HIIT immediately post exercise. These results suggest that high-intensity exercise can stimulate acute myokine responses, with HIIT potentially eliciting a more pronounced signal through IL-6, which may reflect greater muscle activation or metabolic stress [40]. This greater IL-6 response may, in part, reflect a heightened catecholamine surge [41], as high-intensity exercise is known to trigger increased adrenaline and noradrenaline release, which can modulate immune function and enhance IL-6 secretion from contracting muscle, potentially contributing to cancer cell suppression [19].

Previous studies investigated the effects of a single bout of MICT or HIIT on breast cancer cells [25, 29] with suppressed proliferation and viability of MCF-7 and MDA-MB-231 cells in vitro by 12.1 to 24.9% and 10 to 19%, respectively; however, these studies were conducted in apparently healthy individuals, limiting their translation to clinical practice. As previously mentioned, only one study (i.e., Dethlefsen et al. [26]) found inhibitory effects of acute exercise-conditioned serum on MCF-7 and MDA-MB-231 cells in vitro in patients with breast cancer undergoing chemotherapy. Therefore, findings from our study are of clinical relevance as they provide greater insight into the effects of RT and HIIT on cancer cell growth in survivors of breast cancer. Both the RT and HIIT groups showed that growth of MDA-MB-231 cancer cells were significantly inhibited to a similar extent, when applying exercise-conditioned serum collected immediately post as well as 30 min post the exercise session. In addition, when examining the differences between groups, survivors of breast cancer in the HIIT group had significantly greater inhibitory effects on MDA-MB-231 cell growth compared to the RT group immediately post exercise. In our recent review we highlighted that a single bout of moderate to high intensity exercise can have cancer-suppressive effects [19], although research was lacking regarding which exercise mode was more effective in inducing such inhibitory effects. Findings from the current trial indicate that HIIT exerts greater cancer cell suppression compared to RT, although the underlying reasons are unclear. It may well be that the acute effects of exercise-induce myokines on cancer cell growth are influenced by the physical and physiological demand of the exercise session [19]. While both exercise sessions were balanced in terms of duration (i.e., 45 min) and intensity (i.e., ≥ 80% 1RM, 70 to 90% HRmax, RPE 7 to 9), the metabolic equivalent of task (MET) differed, with HIIT reaching 8 to 10 METs and RT 5 to 6 METs [42]. To further support this, serum concentrations of IL-6 were elevated in favour of the HIIT group which, again, may underscore that higher physiological demand may play a key role in skeletal muscle-secreted factors with anti-tumour effects [16].

From a practical perspective, our findings are noteworthy, as regardless of the exercise mode, a single bout of exercise at moderate to high intensity can reduce the growth of MDA-MB-231 cells (i.e., triple negative cancer cells that lack hormone receptors and are therefore not expected to respond to hormonal fluctuations typically influenced by exercise) in survivors of breast cancer, potentially contributing to a lower risk of recurrence. This highlights the importance of exercise as an accessible, non-pharmacological strategy with promising anti-cancer effects, in addition to its physical and psychological health benefits for breast cancer survivors. Clinically, this is relevant because the choice of exercise mode should consider the distinct physical and physiological adaptations induced by RT and HIIT on other outcomes (e.g., body composition, muscle strength, and cardiorespiratory fitness) [8]. This understanding can help guide more tailored exercise prescriptions for breast cancer survivors. Future research should examine the effects of long-term exercise interventions to determine whether these acute myokine responses translate into sustained anti-cancer benefits and improved clinical outcomes.

### Strength and limitations

This is the first study to investigate not only the distinct and independent effects of different exercise modes on anti-cancer myokines such as decorin, IL-6, OSM, and SPARC in survivors of breast cancer, but also in exploring the effects of such exercise modes on MDA-MB-231 cell growth in vitro. In addition, the strength of our study is the relatively larger sample size compared to previous studies using the same approach (participants included ranged from 7 to 20), as well as the inclusion of three distinct time points, allowing for a more comprehensive understanding of the exercise-induced changes over time. In addition, this is the first study to adopt a randomized single-bout design to compare the effects of two distinct exercise modes (i.e., RT and HIIT). This contrasts with previous studies that used single-arm designs (i.e., studies not comparing different interventions), which may limit the understanding of exercise-conditioned serum and its clinical relevance [25, 26, 29, 30]. However, this study is not without limitations. We employed one single breast cancer cell line (i.e., MDA-MB-231), which may limit its applicability to other breast cancer cell. In addition, while in vitro analyses provide insights into the biological association between exercise and cancer growth, culturing cancer cells with exercise-conditioned serum in a 2D environment cannot fully replicate the complex 3D tumour structure in vivo, which influences cell behavior and responses to stimuli [30]. Consequently, 3D cell culture models (e.g., spheroids, organoids) have recently gained interest, as they better mimic in vivo tumour masses, allowing for more accurate assessments of cancer-cell responses to exercise-conditioned serum [43, 44]. However, to date, no acute studies have been performed using such an approach, highlighting the need for future investigations. In addition, in vitro models do not fully replicate the complexity of the physiological environment, including immune system interactions, blood flow, and oxygen availability, which also play a key role in tumour suppression [45–47]. Detailed information on medications that may influence myokine or immune responses (e.g., beta blockers or corticosteroids) was not collected, limiting our ability to account for their potential effects. Lastly, our studies measured a limited array of molecular changes, limiting insights into other underlying mechanisms, including potential contributions from anti-cancer immune function and other exercise-induced circulating factors not assessed in our study. Previous research has shown that exercise-conditioned serum also contains a complex mixture of cytokines, myokines, and hormones that can influence tumor biology beyond that examined in the current study [48].

## Conclusion

This is the first study to examine the acute effects of RT and HIIT on anti-cancer myokine expression and breast cancer cell suppression in survivors of breast cancer. We found that both exercise modes significantly increased myokine levels after the exercise session and there were significant cancer-suppressive effects on MDA-MB-231 cells. In addition, in the between group analyses, there were significant changes in favour of HIIT when measuring IL-6 levels and the inhibitory effects on cancer cells immediately post exercise. Clinically, regardless of the exercise mode, a single bout of exercise at moderate to high intensity can reduce the growth of MDA-MB-231 cells in vitro in survivors of breast cancer, potentially contributing to a lower risk of recurrence. This highlights the importance of exercise as an accessible, non-pharmacological treatment with promising anti-cancer effects.

## Data Availability

The data that support the findings of this study are available from the corresponding author upon reasonable request. Personal data are securely stored in a restricted-access online system at our institute, ensuring compliance with privacy and data protection regulations.
